# Effect of endocrown design modification on retention & microleakage: an in vitro study

**DOI:** 10.1186/s12903-026-09023-w

**Published:** 2026-06-30

**Authors:** Zina Ali Mohamed Anwar, Cherif Adel Mohsen, Rasha Sayed Asaad

**Affiliations:** 1 Fixed Prosthodontics Department, Faculty of Dentistry, Minia University, Minia, Egypt; 2 Fixed Prosthodontics Department, Faculty of Dentistry, Benha National University, Obour, Egypt

**Keywords:** Endocrown, Microleakage, Preparation designs, Retention

## Abstract

**Background:**

Recently, conservative approaches in prosthodontics have gained attention, especially with the use of endocrowns that preserve tooth structure and utilize retention through the pulp chamber.

**Aim of the study:**

This study was conducted to evaluate the effect of modifying endocrown preparation design on retention and microleakage using Vita Enamic blocks in mandibular premolars.

**Methods:**

Forty sound human mandibular premolars were treated endodontically and divided into two equal groups (*n* = 20) according to preparation design: group1: conventional design, and group2: with added buccal groove design. Then each group was further subdivided into 2 equal subgroups (*n* = 10) according to intra-radicular extension: subgroup A: 3 mm; subgroup B: 5 mm intraradicular extensions. Each subgroup was further subdivided randomly into 2 classes (*n* = 5) according to the testing procedure. One class (*n* = 5) was subjected to a retention test using a universal testing machine. The other class (*n* = 5) was subjected to microleakage testing by soaking in 2% methylene blue in an incubator at room temperature for 24 h. All samples were assessed using a digital microscope to evaluate the extent of dye penetration and modes of failure.

**Results:**

Retention testing revealed no statistically significant difference between the two preparation designs within both intraradicular extension subgroups. However, increased intra-radicular extension 5 mm significantly enhanced retention compared to 3 mm in both designs (*p* < 0.001). Regarding microleakage, the buccal groove design showed significantly lower values than the conventional design in both subgroups (*p* = 0.015 and *p* < 0.001, respectively). A significant difference between the two extensions was observed in the conventional group only, with higher microleakage in the 3 mm subgroup (*p* < 0.001), while no significant difference was found in the buccal groove group (*p* = 0.189).

**Conclusions:**

5 mm intra-radicular extension significantly improved the retention of the endocrowns, performing better than 3 mm, while the buccal groove design enhanced retention and resulted in lower microleakage.

## Introduction

Endodontic therapy is a frequently recommended approach for managing teeth with pulp involvement. Traditionally, full-coverage crowns have been the preferred choice for restoring endodontically treated teeth due to their ability to offer comprehensive cuspal protection [1]. However, these restorations necessitate the removal of substantial tooth structure, which may represent a clinical limitation. The restoration of such teeth presents significant challenges in dentistry, particularly due to the compromised strength of the tooth following root canal treatment. This weakening results from the removal of decayed tissue, loss of moisture, and diminished structural integrity [2], highlighting the need for reliable and minimally invasive restorations. Although endodontic posts may improve core retention, post space preparation can inadvertently weaken the root, increasing the risk of fracture and iatrogenic complications such as root perforation or excessive heat during post space preparation can lead to periodontal damage [3].

Mandibular premolar teeth are particularly susceptible to complications in restorative procedures. The smaller size of these teeth limits the available surface area for bonding, making retention more challenging [4]. Although internal grooves in endocrowns were introduced to enhance retention [5], they may not always provide effectiveness because of the smaller tooth structure. For optimal retention, these grooves must be meticulously designed to match the tooth anatomy [6]. Additionally, morphological configuration presents biomechanical concerns. The greater crown height relative to width may create a long lever arm effect, increasing risk of adhesive failure and dislodgment under functional loading [7]. Premolars are also facing unique challenges, particularly due to their vulnerability to lateral and non-axial forces compared to molars, which predominantly receive axial loading, thereby increasing cuspal deflection and fracture risk [8].

Beyond coronal morphology, radicular anatomy further complicates the prognosis of endocrown of mandibular premolar. The pronounced cervical construction and comparatively smaller root diameter may adversely affect stress transmission from the coronal restoration to radicular structure [9].

Advancements in adhesive dentistry and innovations in computer-aided design and manufacturing (CAD/CAM), have enabled more conservative restorations for root canal treated teeth with significant coronal loss. Endocrown serve as a monoblock combining the function of post, core, and cuspal coverage. The retention primarily relies on adhesive bonding. A key feature of endocrowns is the extension of the restorative material into the pulp chamber, which increases the bonding surface area between tooth and restoration and enhances retention [10]. They utilize the internal walls of the pulp chamber for anchorage, making them particularly suitable for teeth with significant coronal damage or extensive defects. Furthermore, they are effective for patients with bruxism or unfavorable occlusal relationships [11]. The conservative nature of endocrowns minimizes the need for excessive dentin removal, thus preserving tooth structure while enhancing the resilience of the tooth to fractures and prolongs the functional lifespan of the restoration [12].

Various CAD/CAM restorative materials are available for use in endocrowns. Among them, polymer-infiltrated ceramics such as Vita Enamic have been widely used due to their mechanical compatibility with dentin [13, 14]. In addition, these materials offer protection for opposing teeth by minimizing excessive wear while also allowing for efficient machining by CAD/CAM technology [15].

Several studies have examined the impact of different endocrown preparation depths, hypothesizing that deeper pulpal cavities provide a larger bonding surface and facilitate better load transmission. Additionally, extending the preparation into the radicular area is believed to enhance restoration stability during cementation, particularly in cases of extensively damaged teeth [16, 17]. Other studies have shown that cavity depths ranging from 2 mm to 4 mm result in acceptable marginal and internal gaps within clinical limits. However, some research has indicated that impression accuracy has a greater effect on marginal and internal gaps regardless of the intra-radicular extension of endocrowns, which directly impacts the adaptation of the restoration [18].

Preparation design plays a crucial role in the mechanical and biological performance of endocrown restorations [19]. Modifications to the internal geometry of the pulpal cavity, such as incorporation of internal grooves have been proposed as a strategy to enhance mechanical performance. These grooves may provide additional macromechanical interlocking, potentially improving stress distribution within the tooth restoration complex [20]. The value of auxiliary retentive features has been well documented in teeth with short clinical crowns for instance, the inclusion of proximal boxes significantly increased resistance to dislodgment compared to conventional designs [21]. Notably, even a single box provide a sufficient improvement in retention without the need for multiple additional features [22]. In addition, the incorporation of a buccal groove has been shown to enhance the overall performance of endocrown restoration increasing the internal surface area for bonding and improving geometric stability of the preparation. This modification not only improves fracture resistance but also contributes to greater mechanical performance [23].

Retention and microleakage are critical factors for the success and durability of endocrowns. Endocrown restorations gain macro-mechanical retention through anchorage to the pulp chamber and cavity margins [24]. Microleakage means infiltration of oral fluids and bacteria at the tooth-restoration interface; it can lead to postoperative sensitivity, recurrent caries, and eventually, failure of the restoration [19]. Moreover, the adhesive techniques employed in endocrown restorations help prevent marginal leakage and reduce the risk of microbial penetration from the crown to the root, thereby enhancing the overall success of endodontic treatments [25].

Although some research has explored the impact of pulpal cavity depth on the mechanical performance of endocrown restorations [16, 26–28], limited evidence exists regarding whether specific modifications to the conventional endocrown design influence both retention and microleakage. In particular, the combined effect of incorporating an internal buccal groove and varying intra-radicular extension depths has not been previously evaluated in mandibular premolars restored.

The null hypothesis of this study was that the modification to the conventional design of endocrown would not affect either retention or microleakage.

## Materials

The following materials were used in study (Table 1)

**Table 1:** Materials used in the study

Material	Manufacturing	Country
Dental probe	Guilin Woodpacker Medical	Guilin, China
Woodpecker Ultrasonic scaler	Guilin Woodpacker Medical	Guilin, China
Preppies pumice paste	Whip Mix Corp	Louisville, KY, USA
Ultrasonic cleaner CDYSON CD-4830	Misr Sinai	China
Preparation burs	Mani, Inc.	Tochigi, Japan
Stainless steel H & K files	Mani, Inc.	Tochigi, Japan
Protaper Universal System	Dentsply-Maillefer	Ballagiues, Switzerland
Gutta-percha	Defend size F2	Malaysia
Adseal	Metabiomed	Cheongju, Korea
EDTA cream	MetaBiomed	South Korea
Diamond wheel stone	Mani, Inc., WR-13	Tochigi, Japan
Cylindrical diamond stone	Mani, Inc., TR-12	Tochigi, Japan
Inverted cone bur	Mani, Inc., EX-21	Tochigi, Japan
Parallel-sided flat end stone (size 2)	Mani, Inc., TF-13	Tochigi, Japan
Trios 5 intraoral scanner	3Shape	Copenhagen, Denmark
Exocad Dental system 2022 v.3.1	Exocad GmbH	Darmstadt, Germany
Vita Enamic Blocks	Vita Zahnfabrik	Bad Säckingen, Germany
CORiTEC 350i (5-axis)	Imes-icore GmbH	Eiterfeld, Germany
Vita Enamic polishing set	Vita Zahnfabrik H. Rauter GmbH	Co. KG, Germany
9.5% Hydrofluoric acid Etchant	Bisco Inc.	Schaumburg, IL, USA
Silane agent	Bisco Inc.	Schaumburg, IL, USA
37% Phosphoric acid	MetaBiomed	Cheongju, Korea
All bond Universal	Bisco Inc.	Schaumburg, IL, USA
BisCem Dual-cure adhesive resin cement	Bisco Inc.	Schaumburg, IL, USA
Light cure	DTE Woodpecker	China
Universal testing machine model 3345	Instron industrial products	Norwood, MA, USA
Testing Bluehill Lite software	Instron instrument	Norwood, MA, USA
Image J 1.43U software	National Institute of Health	Bethesda, MD, USA
2% methylene blue dye	Supreme Organisation for Drugs	Germany
Low speed diamond sectioning saw	Edenta	Au, Switzerland
U500x Digital Microscope	Guangdong	China

## Methods

### Sample size calculation

Sample size calculation was conducted to guarantee adequate statistical power to identify significant differences in retention and microleakage between the tested groups. The calculation was performed using the statistical package for social science (SPSS), assuming a two-tailed test with a significance level of 0.05 and statistical power of 80% (1- β = 0.80). Based on the findings of the study by Hassan SM’s (2020) [29], which reported mean values and standard deviations, a large effect size (f = 0.85) for retention outcome was estimated. The effect size used for sample size calculation was based specifically on the retention outcome. Accordingly, a total sample size of 40 samples (20 samples per main group) was determined to be sufficient to identify statistically significant differences.

### Teeth selection and preparation

Forty human mandibular premolars, extracted for orthodontic or periodontal reasons, were collected from patients who provided informed consent in accordance with the Declaration of Helsinki. Teeth were checked visually and examined using a dental probe to ensure they were free of caries, cracks, or old restorations, and then each tooth was thoroughly cleansed of calculus and soft tissues with dental scalers, nylon bristle brushes, and pumice paste with a low-speed handpiece to remove any excess soft tissue. The teeth were then immersed in 5% sodium hypochlorite for 15 min at room temperature for disinfection. All teeth were subjected to ultrasonic cleaning followed by storing in distilled water at room temperature for two weeks prior experimentation to prevent dehydration. The distilled water was changed regularly to avoid contamination [30, 31].

For every tooth, a conservative access cavity was done using a high-speed large diamond bur. Teeth length was measured. Working length was determined radiographically. Endodontic instrumentation was performed using a combination of manual stainless-steel files for initial root canal negotiation, followed by the rotary Protaper system to complete the cleaning and shaping process, utilizing the crown-down technique up to size F2 with EDTA cream [32]. Irrigation was performed with 5.25% sodium hypochlorite throughout the procedures [33]. Canals were dried with sterile paper points and obturated using resin-based sealer and gutta-percha. The quality of obturation was confirmed radiographically [34].

To assist the endocrown preparation and testing operations, the teeth were mounted in acrylic resin blocks using a specially designed cylindrical mold for sample fixation [35]. Teeth were placed up to 2 mm below CEJ to ensure adequate visibility of the restoration margin during construction and final testing. The occlusal surface of teeth was reduced uniformly by 2 mm with a horizontal and flat surface [36], using a diamond wheel, with copious amounts of water to avoid cracking. The margins were prepared as smooth, continuous butt-joint margins, and with uniform wall thickness of 2 mm [6, 37]. The internal walls of the pulp chamber were tapered with an 8-degree divergence using a cylindrical diamond stone to ensure consistent and uniform wall preparation across all samples. The floor of the pulp chamber was flattened using an inverted cone bur. To ensure smooth transitions, the internal line angles were rounded and refined using a finishing diamond stone.

### Grouping

**Fig. 1:**
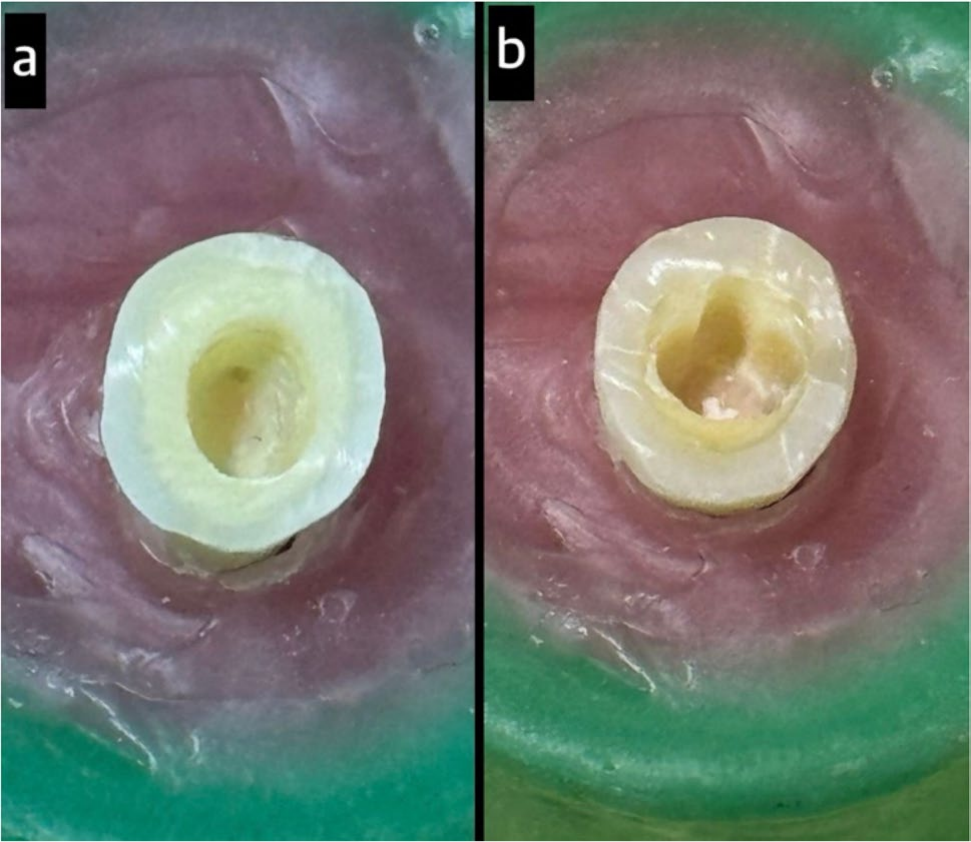
**a** conventional endocrown design; **b** endocrown with added buccal groove design

**Fig. 2:**
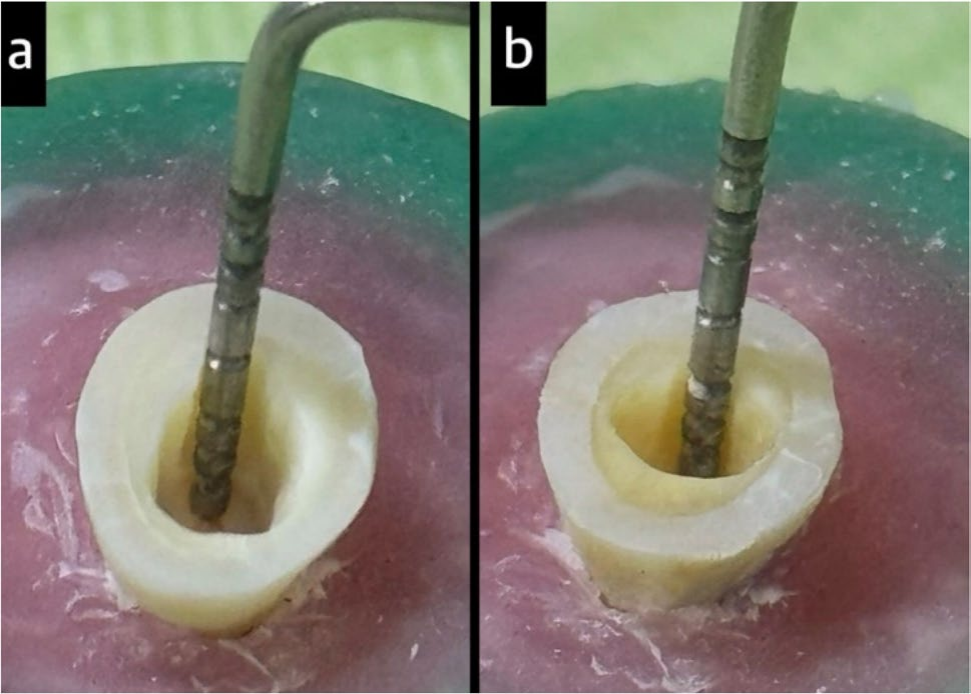
**a** 5 mm intra-radicular extension; **b** 3 mm intra-radicular extension

**Fig. 3:**
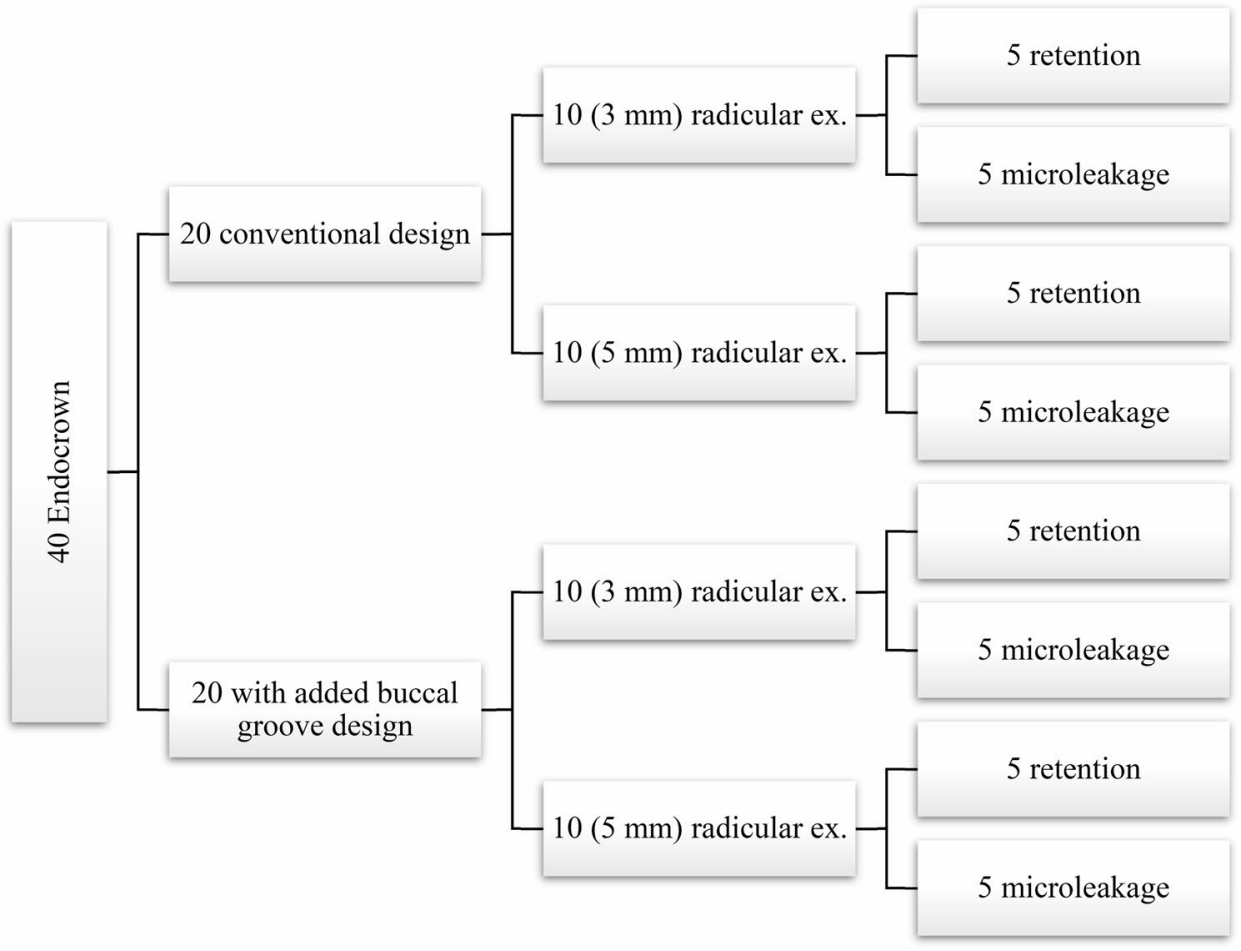
Samples’ allocation and distribution

The samples were divided into two groups: Group 1 was prepared with a conventional endocrown design (Fig. 1a), while Group 2 incorporated an internal buccal groove using a parallel-sided diamond stone with a flat end (Fig. 1b). Each main group was further subdivided into two subgroups based on intraradicular extension. Subgroup A was prepared with a 3 mm intraradicular extension (Fig. 2a), while Subgroup B was prepared with a 5 mm intraradicular extension (Fig. 2b). Both preparations were achieved using the same cylindrical diamond stone, ensuring accurate and uniform cavity preparation to maintain consistency across all samples. Then each subgroup was further subdivided randomly into 2 classes (*n* = 5) according to the testing procedure (Fig. 3).

### Restorations fabrication

**Fig. 4:**
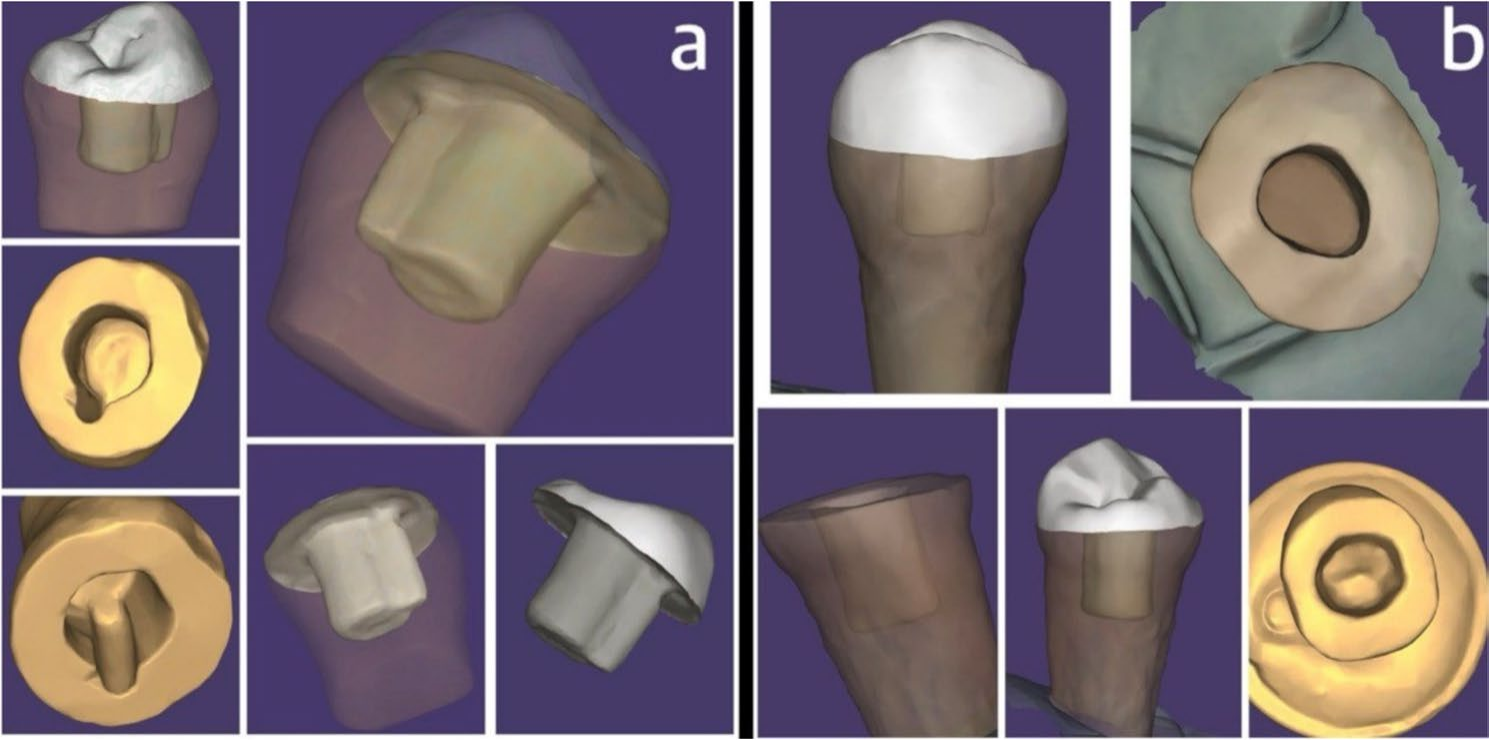
**a** digital designing of endocrowns with buccal grooves & different intraradicular extensions (**b**) digital designing of conventional endocrowns & different intraradicular extensions.

**Fig. 5:**
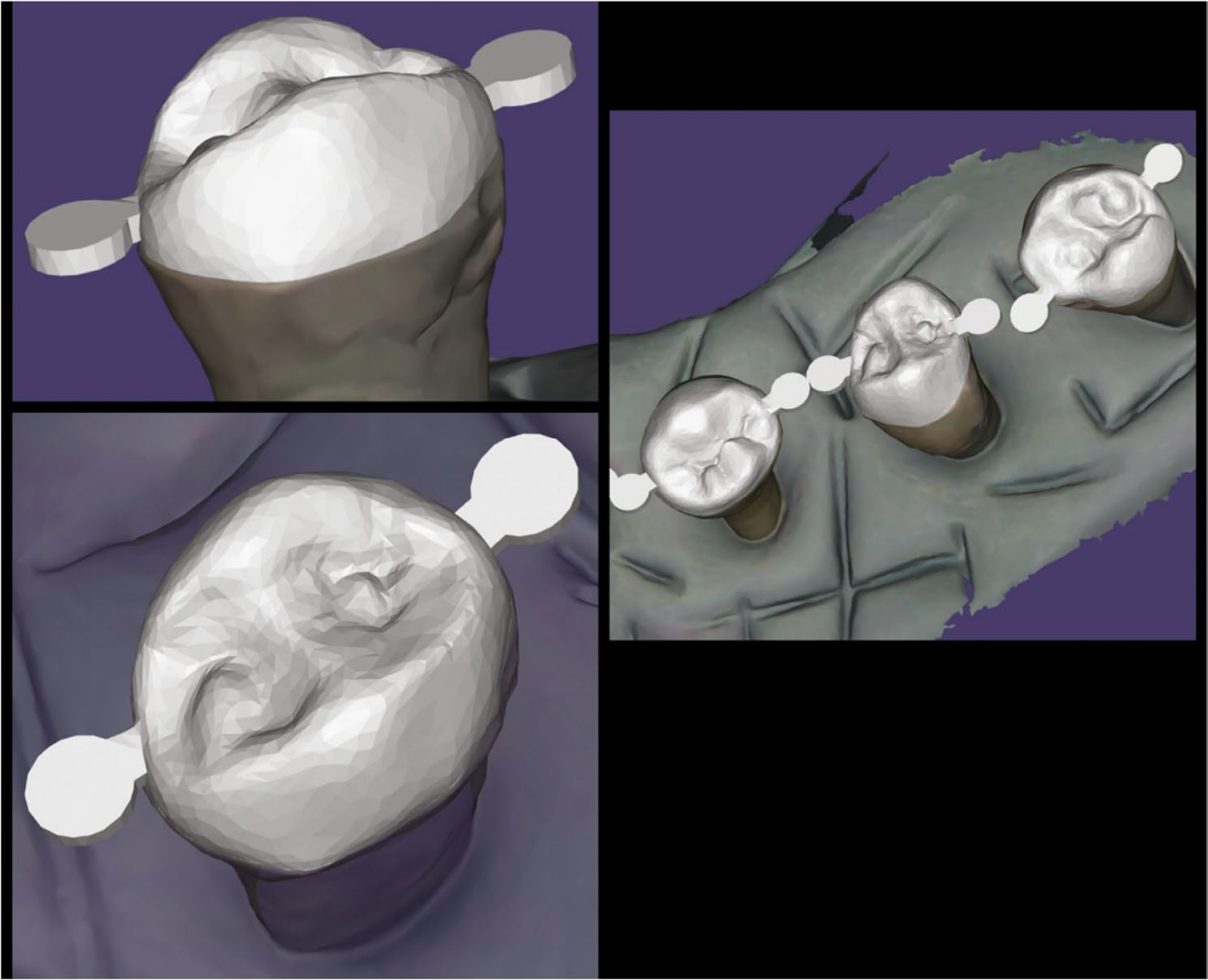
Digital designing showing the lateral projections of the endocrown restoration for the samples that were subjected to retention testing

All premolar teeth were digitally scanned using the Trios 5 scanner, and then the scans were transformed to STL form and sent to the lab. The endocrowns were digitally designed using EXOCAD software [38] (Figs. 4a & b). The samples for retention testing were designed with lateral projections to facilitate their pulling out [39] (Fig. 5). Samples for microleakage testing were designed without these projections. All data were sent to the computer connected to the 5-axis milling machine. Following milling, Vita Enamic endocrowns were polished according to their manufacturer [40].

### Bonding

All restorations were checked for accurate fitting before final cementation. The fitting surfaces of the endocrowns were etched with 9.5% hydrofluoric acid for 20 s, then rinsed [41–44], followed by application of silane coupling agent for 60 s [42], and then allowed to air dry. The prepared tooth surfaces were etched with 37% phosphoric acid for 30 s, rinsed, and dried with compressed air devoid of oil [44]. A light-cure adhesive bonding agent was applied, and allowed to sit for 30 s [45], air-thinned and light-cured for 20 s [46]. An equal amount of dual-cure adhesive resin cement was applied to each endocrown’s fitting surface. Each restoration was seated on its respective tooth [47]. Light curing was initiated for two seconds, followed by axial loading of 5 kg of force for 5 min [29]. Excess resin cement was carefully removed with a scaler, and final light curing was completed for 40 s on each surface [48]. All procedures were performed by the same operator to ensure consistency and reduce the operator’s variability.

### Tests

Retention Testing, each sample was securely fixed in the lower section of a universal testing apparatus controlled by computer software, and the restoration was attached to the upper movable compartment using an orthodontic wire loop (0.7 mm), which was threaded through the lateral projections of the restoration that were designed using dental CAD software. A slowly increasing vertical load was applied in a pull-out mode of force at a crosshead speed of 1 mm/min until total dislodgment of the endocrown. The load required for dislodgment was recorded in Newton [39].

### Failure mode analysis

Fractured samples were cleaned ultrasonically and examined under a digital microscope at a magnification of 40X to analyze their failure mode. The images were transferred to a computer equipped with an image analysis software program. Two types of failure modes observed: adhesive (at the tooth-restoration interface) and cohesive (fracture of tooth structure or endocrown).

Microleakage Testing, measurement was performed by sealing the root surfaces of all teeth with wax, and the entire external surface of each tooth except for a 1 mm zone surrounding the restoration margins was coated with two layers of nail varnish. The samples were then soaked in 2% methylene blue in an incubator at room temperature for 24 h. Subsequently, the teeth were taken out, washed with water, and mounted onto a special holding device for sectioning. The teeth were sectioned mesio-distally with a low-speed diamond saw under water spray and gently dried with tissue paper. Then the dye penetration along the cavity wall, including axial and gingival margins, was assessed using a digital microscope at 35x magnification. Images were captured and transferred to a computer equipped with an image analysis software program, where the leakage was assessed in mm [39].

### Statistical analysis

Version 25 for Windows IBM^®^ SPSS^®^ Statistics was used to provide data into computer. Quantitative data were described using mean and standard deviation for normally distributed data, using Kolmogorov-Smirnov and Shapiro-Wilk tests. An independent t-test was used to compare between two groups in non-related samples or normally distributed data. Two-tailed ANOVA was used to test the interaction between different parameters. The significance of the obtained results was determined using a 5% significance level.

## Results

### Retention

The results of the retention testing, recorded in Newtons, were presented in Table 2. Statistical analysis revealed no significant differences between Group 1 (conventional design) and Group 2 (buccal groove design) within both subgroups: Subgroup A (3 mm intra-radicular extension) & Subgroup B (5 mm intra-radicular extension) (*p* = 0.134 and *p* = 0.061, respectively). In contrast, a significant difference was recorded between Subgroup A (3 mm intra-radicular extension) and Subgroup B (5 mm intra-radicular extension) within both Groups: Group 1 (conventional design) & Group 2 (buccal groove design) (*p* < 0.001). Whereas Subgroup B (5 mm intra-radicular extension) significantly recorded higher retention than Subgroup A (*p* < 0.001). Two-way ANOVA (Table 3) showed that both preparation designs and intra-radicular extensions had a statistically significant effect on retention (*p* < 0.05), while their interaction was not statistically significant (*p* > 0.05).

**Table 2:** Descriptive statistics of retention (Newton) across all groups and subgroups

Variables	Group 1	Group 2	*P*-value^1^
	Mean ± SD	Mean ± SD	
Subgroup A (3 mm)	43.6500 ± 3.5240	51.3100 ± 3.3769	0.134
Subgroup B (5 mm)	71.1500 ± 1.9420	79.6300 ± 3.7742	0.061
*P*-value^2^	< 0.001*	< 0.001*	

*SD* Standard deviation

*P*-value^1^: comparison between Group 1 and Group 2

*P*-value^2^: comparison between Subgroup A and Subgroup B

*Statistically significant difference at *p*-value < 0.05

**Table 3:** Two-way ANOVA assessing the effect of preparation design and the intra-radicular extension on retention

Variables	Mean Square	F	*P*-value
Preparation designs	651.249	6.226	0.017
Intra-radicular extensions	7789.681	74.474	0.000
Intra-radicular extensions X preparation designs	1.681	0.016	0.900
Corrected Model	2814.204	26.906	0.000

F: for a one-way ANOVA test

*P*-value: for comparing the studied groups

*Statistically significant difference at *P* ≤ 0.05

### Microleakage (mm)

The result of the microleakage evaluation measured in millimetres (mm) was shown in Table 4 and revealed that there was a statistically significant difference recorded between Group 1 (conventional design) and Group 2 (buccal groove design) (*p* < 0.001). Moreover, there was a statistically significant difference between Subgroup A (3 mm intra-radicular extension) and Subgroup B (5 mm intra-radicular extension) in Group 1 (*p* < 0.001). In contrast, there was no statistically significant difference between Subgroup A and Subgroup B in Group 2 (*p* = 0.189). Across both Subgroups A & B, Group 1 (conventional design) significantly registered higher microleakage than Group 2 (buccal groove design) (*p* < 0.001 and *p* = 0.015, respectively). Two-way ANOVA (Table 5) showed that both preparation design and intra-radicular extension significantly influence microleakage values (*p* < 0.05). And there was a statistically significant interaction between the two variables (*P* < 0.05).

**Table 4:** Descriptive statistics of microleakage (mm) across all groups and subgroups

Variables	Group 1	Group 2	*P*-value^1^
	Mean ± SD	Mean ± SD	
Subgroup A (3 mm)	0.2284 ± 0.0106	0.0195 ± 0.0057	< 0.001*
Subgroup B (5 mm)	0.1033 ± 0.0159	0.0401 ± 0.0132	0.015*
*P*-value^2^	< 0.001*	0.189	

*SD* Standard deviation

*P*-value^1^: comparison between Group 1 and Group 2

*P*-value^2^: comparison between Subgroup A and Subgroup B

*Statistically significant difference at *p*-value < 0.05

**Table 5:** Two-way ANOVA assessing the effect of preparation design and the intra-radicular extension on microleakage

Variables	Mean Square	F	*P*-value
Preparation designs	0.093	129.905	0.000
Intra-radicular extensions	0.014	19.160	0.000
Intra-radicular extensions X Preparation designs	0.027	37.247	0.0000
Corrected Model	0.044	62.104	0.000

F: for a one-way ANOVA test

*P*-value: for comparing the studied groups

*Statistically significant difference at *P* ≤ 0.05

### Microleakage assessment

**Fig. 6:**
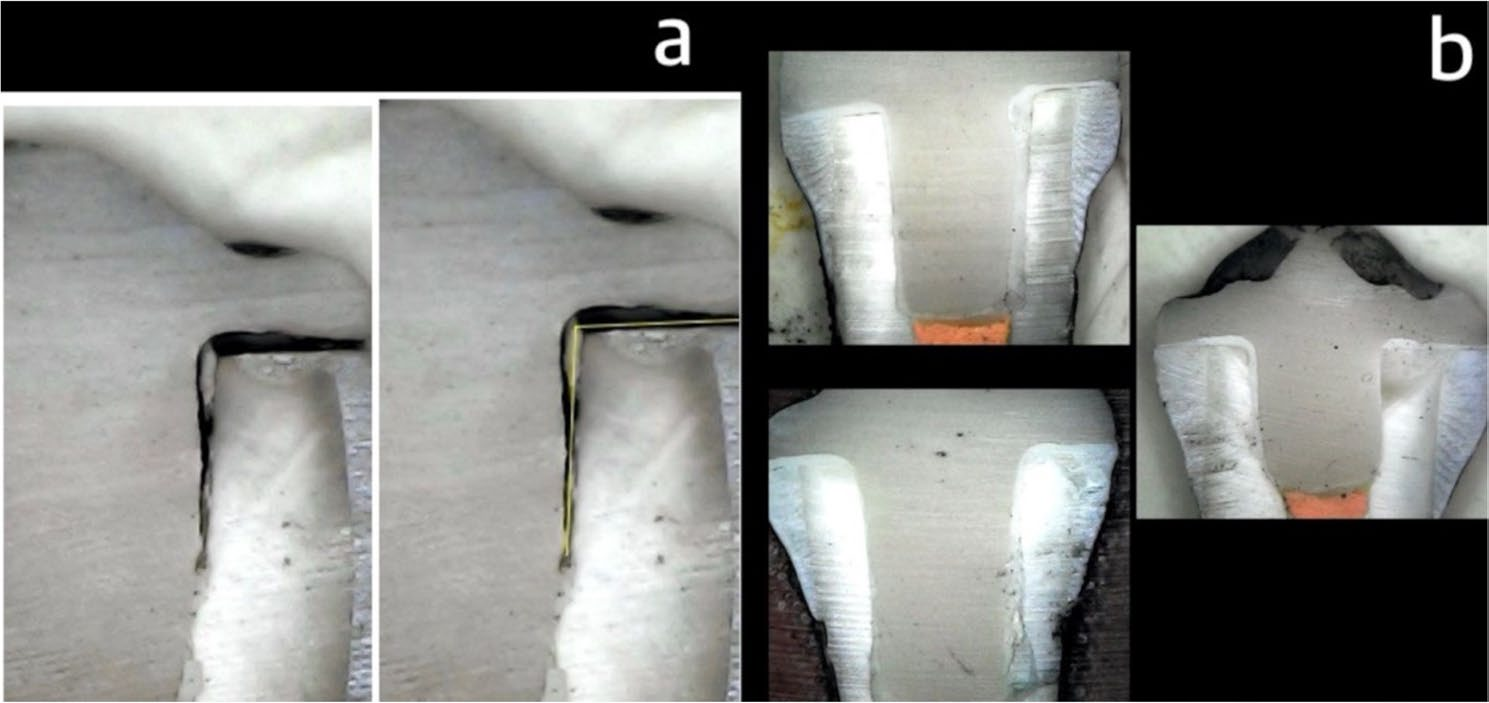
**a** Stereomicroscopic view of endocrown section showing microleakage in conventional design. The yellow line represents the extent of dye penetration. **b** Stereomicroscopic evaluation of microleakage in endocrowns with buccal groove design showing no or minimal dye penetration

Following the samples’ sectioning, the extent of dye penetration was evaluated under a stereomicroscope; the conventional design showed greater dye penetration at both intra-radicular extensions (Fig. 6a), with the highest microleakage observed in Subgroup A (3 mm intra-radicular extension). In contrast, the endocrowns with a buccal groove design exhibited no or minimal microleakage at either 3–5 mm intra-radicular extensions (Fig. 6b).

### Modes of failure

**Fig. 7:**
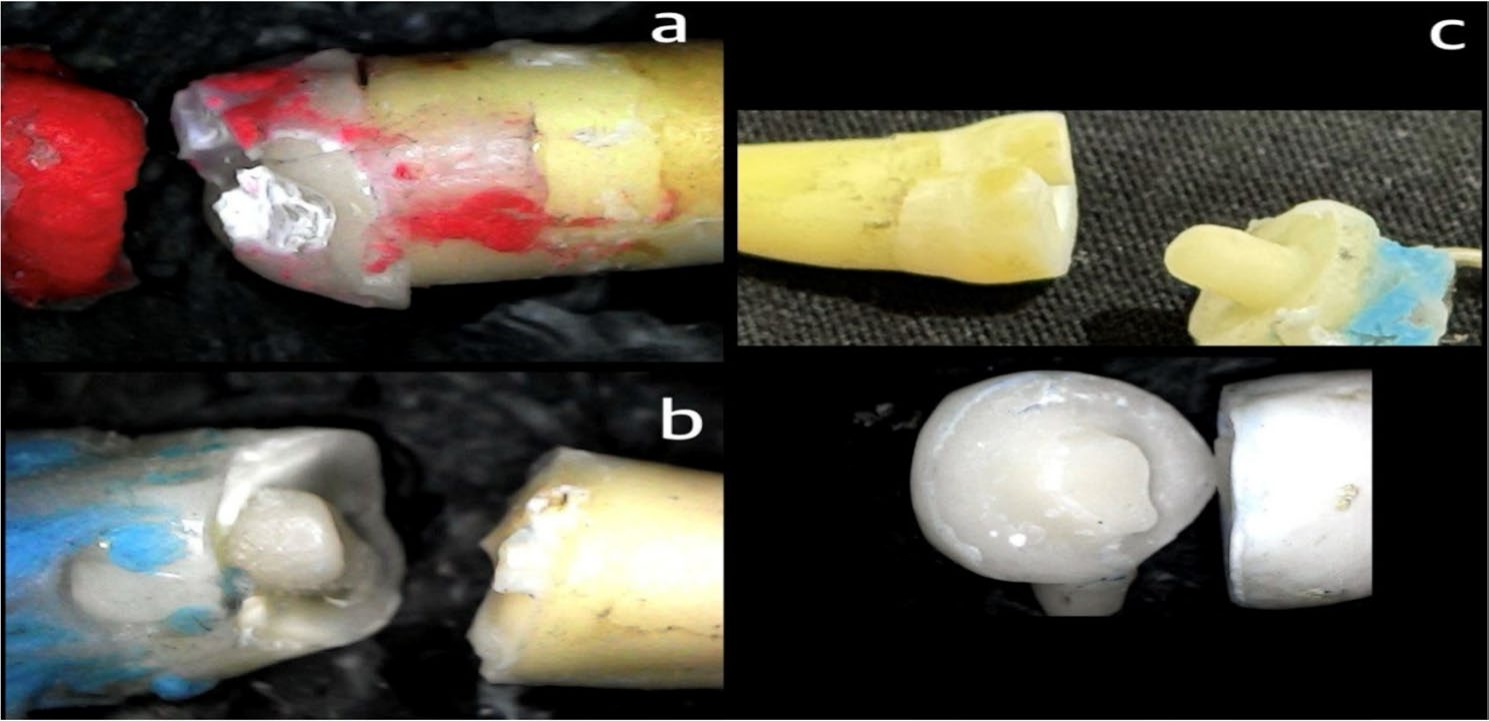
**a** fracture of endocrown restoration (cohesive fracture) (**b**) fracture of tooth structure (cohesive failure) (**c**) dislodgment of the endocrown (adhesive failure)

The samples showed that 5 mm intra-radicular extension required significantly higher retentive force to dislodge the endocrown restoration in both preparation designs compared to the 3 mm intra-radicular extension. This was accompanied by a higher incidence of cohesive failures, particularly in buccal groove design, where fractures were observed either within the restoration (repairable) (Fig. 7a) or involving the tooth structure (unrepairable) (Fig. 7b). The 3 mm intra-radicular extension showed lower retention values and a higher occurrence of adhesive failures, with complete dislodgment of the restorations, especially in the conventional design (repairable) (Fig. 7c).

## Discussion

This study was conducted to evaluate the effect of modifying endocrown preparation design on retention and microleakage using Vita Enamic blocks in mandibular premolars. Restoring endodontically treated teeth with extensive structural loss remains a clinical challenge, as the removal of tooth structure compromises mechanical integrity and long term prognosis [49]. Conventional post-core system may further weaken the tooth and increase risk of microleakage due to multiple bonding interfaces and material mismatch [50–52]. Accordingly, preparation design has a significant influence on the success of endocrown, particularly the intraradicular extension, which enhances retention by increasing bonding surface area. This is especially critical in premolars, where limited pulp chamber size and height crown-to-root ratio [7, 8] necessitate modification in cavity geometry, including the use of internal grooves to improve retention [53].

With the advancement of CAD/CAM technologies, polymer-infiltrated ceramic materials such as Vita Enamic have gained attention due to their unique combination of strength and flexibility, closely mimicking the mechanical properties of natural dentine. This dual-phase structure allows for improved stress distribution and absorption compared to conventional ceramics [39]. In addition it’s microstructure promotes strong bonding with resin-based adhesives, contributing to improve retention and long term performance [54]. This is in agreement with Taha et al. (2018) [55], who reported superior fracture resistance of Vita Enamic even at increased intraradicular extension up to 6 mm, this enhancement was attributed to its favorable internal stress distribution and dual-phase structure.

In this study, the samples were divided according to preparation design into conventional and buccal groove designs. Each group was further subdivided based on intraradicular extension into 3 mm and 5 mm [36]. Each subgroup was equally allocated for evaluation of retention and microleakage. As a result, the null hypothesis was partially accepted. For retention, no significant difference was observed between both preparation designs, but there was a statistically significant difference between both intra-radicular extensions. The results confirm that (5 mm) intra-radicular extension provides higher retention than (3 mm), regardless of the preparation design. For microleakage, a significant difference was found between preparation designs. Also, for intra-radicular extensions, a statistically significant difference was found between 3 mm and 5 mm intra-radicular extensions in the conventional group. But, no statistically significant difference was observed between both intra-radicular extensions in the buccal groove group.

Analysis of the results indicates that the buccal groove design enhances performance of endocrown in terms of both retention and microleakage when combined with pulp chamber anchorage. This aligns with the findings of Al-Dabbagh et al. (2021) [56], who reported that the design not only minimizes the risk of debonding but also ensures a more even distribution of occlusal forces, making endocrowns a durable and conservative option for restoring structurally compromised teeth [9]. Similarly, Biacchi and Basting (2012) [57], demonstrated that modifying the endocrown design by incorporating additional projections into the canals can greatly enhance the retention compared to conventional crown retained by post. These findings were also supported by Gulec and Ulusoy (2017) [58] who showed that modified endocrown designs, where premolar endocrowns were prepared by reducing all walls 2 mm above CEJ, leaving the buccal wall with reduced cusps only, along with additional intracanal extension. This design was found to reduce the amount of stress transmitted to the tooth structure, suggesting that the modified areas contribute to increasing the restoration’s overall thickness and volume. This added bulk allows for better distribution of functional stresses and provides protection for remaining, extensively damaged tooth structure.

Furthermore, combining increased intra-radicular extension with the addition of a buccal groove greatly enhanced retention, likely due to improved mechanical interlocking. These observations were aligned with Mostafavi et al. (2022) [19], who reported that modified preparation designs that include incorporating of grooves or vents, along with deeper extensions, improved the retention of endocrowns. However, they also reported that these modifications can increase marginal discrepancies and compromise the adaptation of endocrowns, thereby increasing the risk of microleakage. This might be attributed to the fact that such modifications complicate the milling process and that additional preparations may compromise the integrity of the remaining tooth structure. In contrast, this present study demonstrated the opposite effect that modified buccal groove design actually reduced microleakage at both 3 mm and 5 mm intraradicular extensions. These findings agreed with Jalali et al. (2023) [59], who compared a conventional endocrown design with 3 mm intraradicular depth with two modified designs: one preserving the buccal wall and the other preserving both buccal and mesial walls. Their findings demonstrated that conventional design showed poorer adaptation and greater axial discrepancies compared to both modified designs. They concluded that preservation of at least one axial wall may improve restoration adaptability and reduce the risk of microleakage. But this was not in agreement with Wakwak et al. (2023) [60], who investigated both conventional and modified endocrown designs, in which the preparation involved retaining the buccal wall intact. Their findings indicated that the modified design exhibited a higher incidence of microleakage than those of conventional preparation. These outcomes could be attributed to several factors. First, the increased complexity of modified designs could affect the marginal seal, making them more susceptible to microleakage. The scanning process used in creating these designs might also fail to achieve an optimal fit in tight areas, resulting in gaps at the margins. This suggests that the complexity of design, combined with potential inaccuracies in scanning, might be the reason for the increasing microleakage observed in modified designs.

Regarding the effect of intraradicular extension, the findings were consistent with Mahgoub et al. (2023) [61], who found that a deeper endocrown with 6 mm intraradicular extension demonstrated higher retention than those with 4 mm intraradicular extension. This may be attributed to the increased surface area available for adhesion and better transmission of functional forces. In addition, Dartora et al. (2018) [62] found that the greater intraradicular extension, particularly at 5 mm, provided higher fracture resistance and greater mechanical stability compared to 3 mm and 1 mm extensions. Similarly, Papalexopoulos et al. (2021) [7] found that a 5 mm intra-radicular extension endocrown exhibited higher retention compared to a 1 mm intra-radicular extension, as the shorter extension was more susceptible to rotation during function. The current results were also aligned with Mously et al. (2025) [50]; who demonstrated that increasing the intra-radicular extension of the endocrown to 5 mm enhanced retention compared to 3 mm and conventional post-core and crown restorations. This could be explained by the same underlying mechanism of increased adhesion surface area associated with deeper antraradicular extension [63]. Conversely, Madani et al. (2023) [64] compared 2 mm and 4 mm endocrown cavity depths for two designs: ferrule design and conventional butt joint (no ferrule) design. They demonstrated that 2 mm cavity depth with a ferrule provides better retention than 4 mm cavity depth. Moreover, the 2 mm cavity depth with a ferrule also outperformed the conventional endocrown design with a 4 mm cavity depth and no ferrule. This might be attributed to the 2 mm cavity depth allowed for preservation of a substantial portion of the remaining tooth structure, which contributed to supporting endocrown restoration. Additionally, the ferrule could have played a compensatory role in enhancing the retention. This is also supported by El-Damanhoury et al. (2015) [65], who concluded that 2 mm intra-radicular extension is sufficient to ensure adequate retention and mechanical stability of the endocrown by the remaining tooth structure and effective reinforcement of weakened tooth structure.

Statistical analysis confirmed that the microleakage observed between the 3 mm and 5 mm intra-radicular subgroups was statistically significant (*p* < 0.05) in the conventional group. The 3 mm intra-radicular extension subgroup exhibited more microleakage compared to the 5 mm intra-radicular extension subgroup within the clinically acceptable limit (≤ 0.64 mm) [66]. This aligns with the study of Hilaly et al. (2024) [67], which reported that the differences in the vertical marginal gap between 2 mm and 4 mm intra-radicular extensions of endocrowns were within clinically acceptable limits. Also, they recommended the 4 mm extension as the more favorable choice for the longevity and durability of the restoration and emphasized that minimizing gaps reduces the risk of bacterial leakage and recurrent caries. Our findings are further supported by the results of Altinci et al. (2025) [68], who found that 4 mm intra-radicular extension resulted in better internal and marginal fit compared to 2 mm and emphasized that accurate fit helps to reduce microleakage and enhance the long-term durability of endocrowns. In support of this, Sudha et al. (2020) [69] demonstrated that an endocrown with a 4 mm intra-radicular extension exhibited less microleakage compared to a conventional post-core and crown restoration when restoring endodontically treated teeth. However, these current results are not in accordance with Ardekani et al. (2024) [18] and Elagwany et al. (2025) [70], who reported that increased intra-radicular extension led to greater discrepancies, attributing this to the fact that areas further from the scanner are less accurate and couldn’t be easily detected. Moreover, the findings of our study are not in agreement with Ghajghouj and Taşar-Faruk (2019) [71], who found that a shallower preparation depth (2 mm) resulted in lower microleakage compared to a deeper preparation (3 mm). These discrepancies might be attributed to factors such as the accuracy of scanning, the milling process of restoration, and material shrinkage which can affect the overall accuracy of restoration. This was also supported by Shin et al. (2017) [17], who compared 2 mm and 4 mm endocrown cavity depths in terms of marginal and internal discrepancies and concluded that 2 mm resulted in lower internal and marginal discrepancies than the 4 mm cavity depth, indicating better adaptation. A potential reason for this could be that the intraoral scanner has limited ability to accurately capture deep areas within the cavity, suggesting the existence of what is known as the shadow phenomenon, which refers to the mesial or distal shadow effect, where a shadow is formed distal to the scanned object, and its extent increases with cavity depth, thereby reducing scanning accuracy.

Our findings showed no statistically significant difference between the 3 mm and 5 mm buccal groove groups, with the lowest microleakage observed in the 3 mm intra-radicular extension subgroup. These findings were in agreement with Darwish et al. (2017) [72], who concluded that the cavity depth alone does not have a considerable influence on the internal structure of the endocrown. In their study, they evaluated 3 mm and 5 mm depths with increased degrees of divergence. The results showed no significant difference between the two depths; however, the modified preparation design improved internal fit and reduced internal gaps, possibly due to increased divergence of axial walls, which facilitates easier and complete seating by reducing mechanical resistance during insertion and enhancing internal adaptation.

However, several studies have highlighted potential limitations associated with deeper intraradicular extensions, particularly in relation to their potential influence on microleakage. This was reported by Giantantzopoulou and El-Damanhoury (2016) [16] who found that the greater intra-radicular extension 5 mm improved retention but resulted in larger marginal and internal discrepancies. It has been suggested that the deep preparations might compromise the accuracy of the captured image during intraoral scanning, potentially leading to blurring of the scanned image, which can lead to inaccuracies in milled restoration. Although these discrepancies negatively affect marginal adaptation, they remain within clinically acceptable limits. Additionally, Alwadai et al. (2023) [73] also confirmed that increasing the intra-radicular extension enhances the retention but negatively affects the marginal adaptation. This could be attributed to increased chances of scanning inaccuracies in deep narrow areas, as well as incomplete seating of restoration due to friction resistance. These issues can lead to stress concentration at the margin during function, which may result in marginal gaps and eventually microleakage.

In our study, cohesive failure occurred in both preparation designs. However, a higher incidence was observed in the 5 mm intra-radicular extension subgroups, especially in the modified buccal groove design. This came in accordance with Ghoul et al. (2020) [20], who compared a conventional design to a modified design incorporating mesial and distal grooves and found that the modified design exhibited significantly greater retention. Most of the failures observed were cohesive, indicating that the modified design offered enhanced retention of restoration. Unlike the findings reported by Zardoni et al. (2023) [74], who found conventional endocrown preparation designs considered sufficient to maintain adequate retention of restoration without the need for additional retention features due to the inherent advantage of their monoblock design, and related their results to that endocrown aims to preserve as much healthy tooth structure as possible.

The present study has several limitations, primarily related to its in vitro nature, which does not fully replicate the complexity of the oral environment. Critical clinical factors, including thermal fluctuations, cyclic loading, masticatory forces, and variations in saliva composition and pH, were not simulated. In addition, thermomechanical aging was not performed, limiting the ability to predict long-term clinical performance and durability. Moreover, the use of static laboratory conditions may not accurately reflect the dynamic intraoral environment. The study was also limited to a single restorative material and cementation protocol, restricting comparisons with alternative materials and bonding strategies.

Within these limitations, the findings provide preliminary evidence regarding the influence of endocrown design on retention and microleakage. The results suggest that incorporating a buccal groove preparation combined with increased intraradicular extension may enhance retention and marginal integrity of endocrowns in mandibular premolars. However, further studies conducted under clinically relevant conditions, particularly those incorporating thermomechanical aging, are necessary to validate these findings and assess long-term effectiveness and reliability.

## Conclusions

### Within the limitations of this in vitro study, it was concluded that

- Intraradicular extension significantly enhanced retention of VITA Enamic endocrown restorations in mandibular premolars, with 5 mm depth showing superior retention compared to 3 mm depth, regardless of preparation design.
- The buccal groove preparation design demonstrated improved retention compared to the conventional design in both intraradicular extensions.
- The buccal groove preparation design was associated with reduced microleakage, suggesting improved marginal seal, while a 5 mm intraradicular extension further decreased microleakage, particularly when combined with this preparation design.

## Data Availability

The datasets generated and analyzed during the current study are available from the corresponding author on reasonable request.

## Abbreviations

CAD/CAM: Computer-aided design & computer-aided manufacturing
ex.: Extension
STL: Stereo-lithography
mm: Millimeter
Min.: Minute
IPM SPSS: Software for Advanced Statistical Analysis
P: Probability
